# A randomized controlled trial of *Explore Transplant at Home* to improve transplant knowledge and decision-making for CKD 3–5 patients at Kaiser Permanente Southern California

**DOI:** 10.1186/s12882-019-1262-9

**Published:** 2019-03-04

**Authors:** Amy D. Waterman, Crystal Anderson, Angelika Alem, John D. Peipert, Jennifer L. Beaumont, Shayna L. Henry, Bhanuja Dub, Lizeth Ambriz, Neha Bijjala, Amanda Faye Lipsey, Brian Mittman

**Affiliations:** 1 Division of Nephrology, David Geffen School of Medicine, University of California Los Angeles, Los Angeles, California USA; 2 Terasaki Research Institute, Terasaki Research Institute, 1018 Westwood Blvd, Los Angeles, CA 90024 USA; 3 Kaiser Permanente Southern California, 100 S Los Robles Ave, Pasadena, CA 91101 USA; 4 Department of Medical Social Sciences, Feinberg School of Medicine, Northwestern University, Chicago, USA

**Keywords:** Kidney transplantation, Living donor, Racial disparities, African-Americans, Hispanics, Patient education, Health knowledge/attitudes, Transtheoretical model

## Abstract

**Background:**

Five-year survival on dialysis is only 40%, compared to 74% with a deceased donor kidney transplant (DDKT) and 87% with a living donor kidney transplant (LDKT). An American Society of Transplantation (AST) Consensus Conference recommended that patients with chronic kidney disease (CKD) Stages 3–5 have the opportunity to learn about and decide which treatment option is right for them, particularly about LDKT. However, early education about LDKT and DDKT outside of transplant centers is inconsistent and often poor, with patients in CKD 3 and 4 and ethnic/racial minorities even less likely to receive it. A new randomized control trial (RCT), in partnership with Kaiser Permanente Southern California (KPSC), will assess knowledge gaps and the effectiveness of a supplementary video-guided, print and technology-based education intervention for English- and Spanish-speaking patients in CKD Stages 3, 4, and 5 to increase LDKT knowledge and decision-making. To date, no published LDKT educational interventions have studied such a large and diverse CKD population.

**Methods:**

In this RCT, 1200 English and Spanish-speaking CKD Stage 3–5 patients will be randomly assigned to one of two education conditions: *ET@Home* or KPSC standard of care education. Randomization will be stratified by CKD stage and primary language spoken. Those in the *ET@Home* condition will receive brochures, postcards, DVDs, and text messages delivering educational content in modules over a six-month period. Baseline data collection will measure demographics, transplant derailers, and the amount of previous CKD and transplant education they have received. Changes in CKD and transplant knowledge, ability to make an informed decision about transplant, and self-efficacy to pursue LDKT will be captured with surveys administered at baseline and at six months.

**Discussion:**

At the conclusion of the study, investigators will understand key knowledge gaps for patients along the CKD continuum and between patients who speak different languages and have assessed the effectiveness of both English- and Spanish-language supplementary education in increasing KPSC patients’ knowledge about the opportunities for and risks and benefits of LDKT. We hope this program will reduce disparities in access to transplant.

**Trial Registration:**

ClinicalTrials.gov Identifier: NCT03389932; date registered: 12/26/2017.

## Background

There are 30 million Americans with chronic kidney disease (CKD) and at risk for kidney failure, and 660,000 patients with end-stage renal disease (ESRD). To sustain life, ESRD patients, 31% of whom are Black, 15% Hispanic, and 5% Asian, must either receive daily or weekly dialysis treatments or have a kidney transplant. The five-year survival rate for patients on dialysis is only 40%, compared to 74% for patients who receive a deceased donor kidney transplant (DDKT) and 87% for patients who receive a living donor kidney transplant (LDKT) [1]. Over 95,000 individuals are awaiting a DDKT in the United States, with more being added daily (based on OPTN data as of 06/08/2018).

Patients who receive kidney transplants live 5–15 years longer than if they remained on dialysis and have a better health-related quality of life (HRQOL), including a greater likelihood of being in the workforce during their critical earning years [2]. While LDKT is the medically optimal and most cost-effective renal replacement therapy (RRT) for patients with ESRD, LDKT rates have declined by 17% from 2004 to 2014. In 2014, in the U.S., 17,106 patients received a transplant (5536 LDKTs) while 8021 patients died or became too ill to remain on the list; 70% who died were racial/ethnic minorities. Of the 2281 transplants performed in California in 2017, only 604 were from living donors (OPTN data as of 06/08/2018).

Providing comprehensive education to CKD patients about the benefits of LDKT as early as possible can increase the chances that they will learn about it, seek living donors, and ultimately receive an LDKT [3]. Many patients, particularly socioeconomically disadvantaged patients and patients of racial/ethnic minority groups [4], have not had sufficient DDKT and LDKT education before their kidneys failed. [5, 6] Generally, patients not considering LDKT lack knowledge about the benefits of living donation over remaining on dialysis [7], have concerns about involving and risking a living donor’s health [7], fear asking others to donate a kidney [8], or fear their own surgical pain and the possibility of the transplanted kidney failing [9].

While Black and Hispanic patients experience higher rates of diabetes and hypertension [5] and increased chances of developing ESRD versus their White or non-Hispanic counterparts [10], they are less likely to complete transplant evaluation [6, 11, 12], experience longer wait-list times [13, 14], and are less likely to receive a DDKT or LDKT [6, 10, 15, 16]. While 11.4% of White patients had received an LDKT after two years of being wait-listed, only 2.9 and 5.9% of Black and Hispanic patients, respectively, had received an LDKT [11, 17]. The disparity in transplant knowledge between White patients and patients of racial or ethnic minorities has been well documented by our research team [7, 8, 18, 19].

Without a nationally coordinated healthcare system in the United States, it is difficult to ensure that patients moving from CKD Stages 3 and 4 to ESRD are consistently making informed transplant decisions. Limited research is available about how transplant education for patients in CKD Stage 3 and 4 is delivered. One study in community nephrologist’s offices, the Talking About Live Kidney Donation (TALK) program, compared the efficacy of an LDKT print and video program, with or without in-person social worker discussions, on CKD patients’ steps toward beginning transplant evaluation [20]. Though the TALK trial found that the discussion-oriented, social worker intervention had a higher predicted probability of taking additional steps in comparison to the education-only group, it also found that significantly higher proportions of patients in the education-only group took key steps towards transplant, like completing transplant evaluation.

Effective strategies for transplant education within dialysis centers are important to understand since 70% of ESRD patients are on dialysis [21], some of whom may never present to a transplant center for evaluation. Our team surveyed dialysis educators at 170 dialysis centers and found that 81% of educators were educating patients by recommending that they learn more about transplant by going to a transplant center or learning about transplant themselves [22]. In another study, only 24% (297 of 1223) of “informed” patients (based on patient-reported data submitted to the Centers for Medicare and Medicaid Services on Form-2728) reported that educators had detailed discussions about the risks and benefits of DDKT and LDKT with them [22]. Other similar studies have found that less than half of dialysis patients receive comprehensive discussions or counselling about the risks and benefits of transplant [22]. Our previous research has also shown that while kidney patients spend over 500 h in the dialysis center annually, they only spend a median of one hour reading brochures about transplant and 30 min talking about transplant with medical staff [4]. Only a minority of dialysis centers have formal education programs or provide transplant education to share with potential living donors [22–24]. For these and many other reasons, evidence suggests that not all dialysis patients receive appropriate information about transplant [5, 6, 22, 23, 25].

Previous research has shown that two of the strongest predictors of successful completion of transplant evaluation are having access to more transplant education resources and having greater transplant knowledge at the onset of transplant evaluation, the latter also being the only significant predictor for ultimately receiving an LDKT [26]. With providers outside of transplant centers reporting constraints to educating patients about transplant [23], it is possible that providing supplementary education within a large health care system, with its fully integrated care management program, may be an opportune way to efficiently educate more CKD 3–5 patients about the opportunities for and risks and benefits of DDKT and LDKT. Further, the use of technology, such as text messaging, videos, and digital applications may reduce the provider burden barrier to delivering transplant education.

There is previous evidence that delivering more comprehensive education over time may be helpful to patients making informed decisions outside of transplant centers. The RaDIANT program in Georgia which targeted dialysis providers and patients with a multi-component educational intervention including patient educational toolkits, videos, and a transplant mentoring program over one-year found a 75% increased adjusted odds of referral for kidney transplant evaluation and that the intervention activities were more effective for Black versus White patients [27]. Through a group randomized controlled trial, a version of *Explore Transplant (ET)* delivered face-to-face over four meetings with patients while they were undergoing dialysis, was shown to increase patients’ knowledge and informed decision-making significantly more than standard transplant education offered in dialysis centers [28]. Finally, *Explore Transplant@Home* (*ET@Home*) delivered by mail and supported through bimonthly postcards and texting over an eight-month period also found significant increases in transplant knowledge and informed decision-making among ESRD patients who received *ET* in this format, compared with standard transplant education in dialysis centers [29].

To date, *ET* and *ET@Home* have never been studied with patients who are in CKD Stages 3 & 4 or with Asian or Spanish-speaking patients. This study is a partnership between UCLA and Kaiser Permanente Southern California (KPSC). KPSC’s fully integrated care management program, diverse membership, and ability to track a large patient population offers a rare opportunity to study how knowledgeable CKD Stage 3–5 patients are about LDKT, assess disparities in knowledge across many races, ethnicities, and languages, and conduct a large-scale trial of the effectiveness of *ET@Home*. This manuscript will describe the protocol for a new randomized control trial (RCT) conducted in collaboration with KPSC to assess the effectiveness of *ET@Home* within a large healthcare system with a diverse group of CKD 3–5 patients.

## Study design

The purpose of this study is to assess whether the *ET@Home* program can be integrated successfully across a large racially, ethnically, and geographically diverse patient catchment area and to assess its effectiveness to increase knowledge about the risks and benefits of LDKT and increase informed decision-making. The effectiveness of the *ET@Home* program will be evaluated in a randomized controlled trial (RCT) of 1200 Black, Hispanic, Asian, and White patients with stage 3, 4, or 5 CKD. Patients will be randomized to receive: (1) no additional education other than what is provided within KPSC (standard-of-care); or (2) a video-guided, four-part *ET@Home* program delivered by mail and supported through bimonthly postcards and texting over six months. The study has two aims.

### Aim 1

Before intervention, to assess differences in knowledge and decision-making about the opportunities for and risks and benefits of living kidney donation for 1200 CKD Stage 3–5 patients by race/ethnicity and primary language spoken.

#### Hypothesis

*Patients earlier in the CKD continuum, patients who speak Spanish, and non-White patients will have less knowledge about the opportunities for and risks and benefits of living kidney donation and will be making less informed LDKT decisions.*

### Aim 2

To conduct a randomized controlled trial in English and Spanish of *ET@Home* for CKD Stage 3–5 patients to assess its effectiveness to increase LDKT knowledge and decision-making by race/ethnicity and primary language spoken as compared to the standard KPSC education.

#### Hypothesis 1

*At the conclusion of the trial, CKD patients who receive ET@Home will have greater transplant knowledge and be more likely to make an informed treatment decision than patients receiving KPSC education alone.*

#### Hypothesis 2

*ET@Home will be more or equally effective for patients earlier in the CKD continuum, patients who speak Spanish, and non-White patients than patients in these subgroups receiving KPSC education alone.*

## Methods

### Theoretical Foundation of ET@home

The *Explore Transplant* (*ET*) education program was created based on Prochaska’s Transtheoretical Model of Behavioral Change (TTM) and research with over 1000 patients with CKD, to address gaps in their transplant knowledge [30]. *ET* allows transplant-eligible patients to explore the option of remaining on dialysis or pursuing DDKT or LDKT and make an informed choice after knowing their benefits and risks. The TTM holds that patients vary widely in their levels of readiness to make important decisions about their health [31], like whether or not to get an LDKT [32]. This study will employ educational resources from the *ET@Home* program that are based on the TTM and that are designed to help CKD patients at all stages of readiness and decision making around DDKT and LDKT learn more about their treatment options and to ultimately choose the option best for them. The *ET@Home* educational resources never pressure patients to pursue transplant of any kind; rather, they provide accurate information about the complex set of potential risks and benefits that need to be considered when deciding whether to get a DDKT or LDKT, including potential increases in length and quality of life, resuming life activities, implications of taking transplant medications, the chances of injury to a living donor, and many more factors.

### Study participants: Eligibility, recruitment, and randomization

KPSC provides coverage and care to nearly 65,000 patients with CKD Stages 3–5 throughout Los Angeles, San Diego, Kern, San Bernardino, Riverside, and Ventura Counties. About 24% of these patients are Hispanic, 52% White, 15% Black, and 9% Asian, similar to the broader CKD population in California. As of December 2015, more than 1500 KPSC CKD patients were listed on the United Network for Organ Sharing (UNOS) waiting list awaiting a DDKT, and approximately 700 additional KPSC patients were undergoing transplant evaluation. However, since 2014, only 350 KPSC patients have received a kidney transplant, with fewer than 100 patients receiving a LDKT.

Inclusion criteria for the study include adult KPSC patients between 18 and 70 years holding continuous membership with KPSC, currently being treated for CKD 3, 4, or 5/ESRD, having had at least one visit to a KPSC nephrologist in the last 18 months and who speak English or Spanish. To target enrollment of the CKD 3 patients at highest risk of worsening CKD, investigators will calculate a risk score for CKD 3 patients using the model described in Tangri et al. [33]

Exclusion criteria include having known medical or other permanent contraindications to transplant including cancer, heart failure, chronic obstructive pulmonary disease, hepatitis, cirrhosis, or dementia, or previous delisting or rejection for renal transplant by a transplant center. Other exclusion criteria include not being able to speak and read English or Spanish or having previously received a transplant. Those who have already refused to be evaluated for a transplant will also be excluded from the study. Patients’ transplant eligibility will be confirmed by reviewing relevant information (e.g., absence of serious heart disease or cancer) from their Kaiser electronic medical record.

**Fig. 1:**
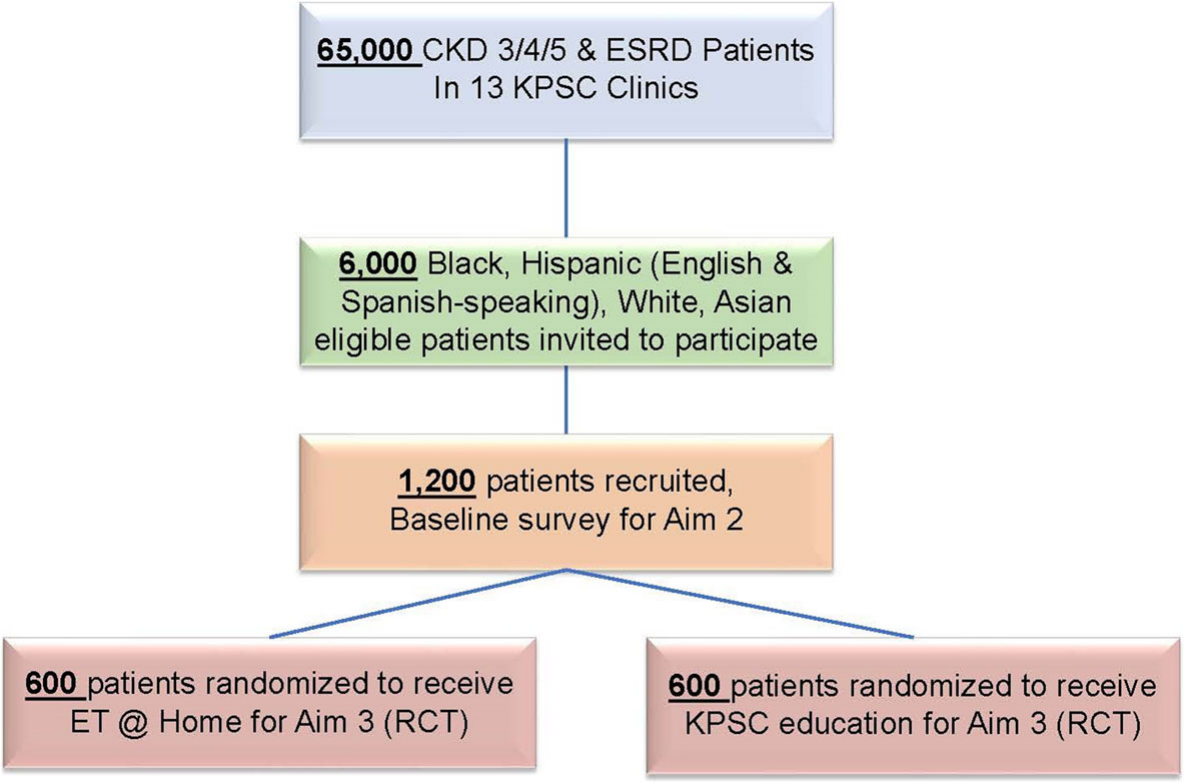
Patient Recruitment Flowchart

Prospective participants who meet eligibility criteria will be sent an email inviting them to enroll in the study and asked to complete the initial transplant decision-making survey online or on the phone. Study staff will invite patients to participate in the RCT, beginning at the top of the risk-score ranked list, moving down the list until stratified cell targets are met. Recruitment will be stratified by race/ethnicity, language spoken, and CKD stage, oversampling smaller subgroups within the KPSC patient population. Expecting a response rate of 20%, an initial sample of 6000 patients will be invited to participate, resulting in a final sample of 1200 patients (Fig. 1). Clicking the survey link or contacting the study team by phone to begin the survey indicates consent to participate in the study. The email includes an opt out link if they do not wish to participate in the study. Invited prospective participants will receive follow-up invitations by secure email and phone to complete the initial transplant decision-making survey.

After completing the initial survey, enrolled participants will be randomized in a 1:1 ratio, stratified by race/ethnicity, language spoken, and CKD stage, to either the *ET@Home* intervention condition or standard KPSC education. The randomization sequence is produced electronically within the data capture system. Once an enrolled participant is assigned to an intervention arm, the study team will send the participant a notification of their assignment. Since this is an educational intervention, neither the study participants nor the study team are blinded to treatment assignment.

### Settings

Since the CKD patients served by KPSC receive care in 13 KPSC centers spanning over 2000 mile^2^ ranging across Los Angeles and San Diego metropolitan areas, an intervention strategy and setting that can reach all of these patients needs to be employed. Also, the intervention needs to reach racial and ethnic minority patients, who often have fewer transportation resources available to them and may be less able to travel even locally to take advantage of CKD education programs. Finally, the DDKT and LDKT education program needs to be flexible and capable of integration into the ongoing transplant education and care management programs within KPSC so it does not create additional burden on busy transplant coordinators and patient educators. Thus, the setting for this *Explore Transplant@Home* trial will be guided learning within the patient’s homes, for patients and any interested individuals in their social support networks, through use of video, print, and text education.

### Intervention (RCT educational components)

#### Description of standard KPSC transplant education (control)

All KPSC nephrology departments currently offer a 1–2 h course in transplant education to all CKD patients interested in pursuing transplant, whether from a living or deceased donor. The KPSC course addresses topics including how kidney transplantation works, determining whether kidney transplantation is the right choice, the steps involved in pre-transplant evaluation and referral to transplant centers of excellence. Because time is limited, the bulk of the content covered is oriented toward developing an understanding of the evaluation and wait-listing process, rather than on the risks and benefits of pursuing LDKT. Patients are encouraged to bring any prospective donors to the class with them, and course attendees learn about the basics of donor testing, some simple information regarding matching and donor exchanges, and reminders that LDKT can result in a much shorter wait for a donor organ. Written materials and presentations from the educators do recommend that patients seek out living donors but do not provide details on how to approach prospective donors. The materials address only the risks and benefits to the recipient not to the donors. Compared to DDKT, much less detailed information is provided regarding LDKT, unless prompted by interested patients. Practical information about waiting for a transplant, including local wait-list times and allocation criteria, the transplant procedure, and steps for post-graft follow-up are covered in detail. KPSC educators include nephrology nurses and social workers. The course is presented monthly in English with available real-time language interpreters at each of 13 local medical centers, in Spanish at 1–2 centers, and is supplemented by a written pamphlet in English and Spanish.

While patients at all phases of CKD and their family members and support persons are welcome to attend the transplant education course, it is primarily focused on individuals who have reached an advanced stage of CKD and/or ESRD and are contemplating renal replacement options, or who are new to dialysis and considering transplant as a renal replacement approach. Patients who wish to be evaluated for transplant eligibility and move forward with the process of pre-transplant assessment and listing are required to complete this course before beginning pre-transplant work-up. For this trial, routine transplant education practices will be continued without any content changes. Patients who are randomized to the control condition and who are or become potentially eligible for transplant will not receive any additional interventions within KPSC during the study period.

#### Description of patient-guided *Explore Transplant@Home* program

Patients in the *ET@Home* study condition will receive four modules of video and print transplant education over a 6-month period (Table 1). The modules sequentially help patients think through what is important to them, learn what it might be like if they became a kidney recipient or involved a living donor, the risks and benefits of each, and, at the program’s conclusion, make a decision about the best treatment option for them. The four videos and their corresponding print brochures – “Exploring Transplant”, “Kidney Recipients’ Transplant Experiences,” “Living Donors’ Donation Experiences,” and “Making an Informed Choice” – discuss in detail the medical, psychological, and financial risks of being a deceased or living donor. These modules cover ways to slow down kidney disease and include real life quotes and stories from people who have stayed on dialysis, received DDKT and LDKT, or been a living donor. The “Making an Informed Choice” brochure is a decision aid that guides patients in choosing a path that is right for them and provides helpful websites that patients can use as resources to learn more. At the conclusion of the program, patients individually choose which renal replacement treatments may be right for them and plan a set of small steps they want to take next.

**Table 1:** *Explore Transplant@Home* Module Content

Module 1: The Basics of Kidney Disease*	General Contents	Brochure	Postcards	Video
	Welcome & Info Card Video #1 (DVD or link)	“Basics of Kidney Disease” o What are kidneys and what do they do? o What is kidney disease? o What are the treatment options for kidney disease? o Why kidney patients get transplant o Why people donate their kidneys	“What is Chronic Kidney Disease?” “How can I slow down kidney disease?” “What are the treatment options for kidney failure?”	“Exploring possible treatment options” This introductory video explores what kidney disease is and what patients can do to slow down kidney disease through different treatment options.
Module 2: What You Need to Know About Kidney Transplants*	General Contents	Brochure	Postcards	Video
	Module 2 Card Video #2 (DVD or link)	“What you need to know about kidney transplants” o Your treatment options: Dialysis or transplant o Your treatment options: Deceased or living donor o Deceased donor kidney transplants o Kidney patient evaluation, surgery, and recovery o Possible risks of a transplant	“What are the advantages of a transplant?” “What are the 2 types of transplants?” “What is transplant evaluation like?”	“Learning more about becoming a kidney transplant recipient” This video compares the benefits and risks of dialysis, deceased donor transplant, and living donor transplant.
Module 3: What You Need to Know About Living Donation*	General Contents	Brochure	Postcards	Video
	Module 3 Card Video #3 (DVD or link)	“What you need to know about living donation“ o Kidney transplants from a living donor o Living donor evaluation, surgery, and recovery o Possible risks to a living donor	“What are the advantages of living donation?” “What is it like to donate a kidney?” “What are the risks of donating a kidney?”	“Choosing between deceased donor transplant and living donor transplant” This video explores the benefits and risks of both transplant surgeries, specifically what people should know about living donation.
Module 4: How to Decide What to Do About Your Kidney Disease*	General Contents	Brochure	Postcards	Video
	Module 4 Card Video #4 (DVD or link)	Brochure: Deciding what to do about your kidney disease o Making a decision about your treatment o Keep thinking about your treatment options o Visit websites to learn more o Choose a treatment option that’s right for you o Your next steps	“What are the pros and cons of the options?” “How can you keep learning more?” “What is the right decision for you?” “What should I do if I am interested in living donation?” “What should I consider before making a decision?”	“Deciding what to do and steps to take” This video explores patients deciding what treatment options is best for them and what steps to take.

*Excludes text message sequence as it is too large for this publication. See Table 2 for an example of the text message sequence

To expand this program for patients in CKD 3 and 4, we conducted a combination of 32 additional focus groups and individual interviews to learn what additional content should be added. Based on recommendations, we added more content about the purpose and function of kidneys, explaining in detail what kidney disease is and its various stages, how to slow down the kidney failure process, and providing an overview of treatment options once the patient enters kidney failure. Experts from the nonprofit organization, Health Literacy Media, and the study investigators reviewed and revised all print content using the most current, evidence-based health literacy principles [34]. The review and edits included considerations around audience appropriateness, readability, behavioral orientation, interactivity, information architecture, plain language, and clear design.

Each *Explore Transplant@Home* video begins and ends with a transplant educator and kidney professional or patient introducing the video and then making specific recommendations afterwards about what to think about next. The healthcare professionals on the video are diverse in their gender and ethnicity. On the final video of the series, a patient and his wife models meeting with the educator to work through the decision-aid and make a decision that is right for him or her. This modeling occurs on the video to support the patient watching and doing the same at home with his family. The Spanish language version of the video (voiced in Spanish, not subtitled) has been awarded a certificate of translation.

During the baseline survey, patients will be asked about the availability of Internet and DVD resources in their home. If the patient does not have a DVD player, a DVD player will be provided as a service of the grant. Should they prefer to watch this program via Internet streaming, an individualized login code will be provided to them. Individual login data for patients utilizing the Internet will be tracked to determine fidelity.

After each module of print and video education is mailed, postcards will be mailed weekly, recapping important content covered within the videos and brochures. Patients will have the opportunity to participate in a texting component of *ET@Home* that also sends educational content through learning reminders and questions by SMS text message each week (Table 2). Study participants will receive text messages daily that include study participation reminders, motivations to continue in the study, CKD facts, recommendations for learning more, and quiz questions to test knowledge. Participation in the text messaging portion of the trial is voluntary, and the option to opt out is available at all times.

**Table 2:** Examples of ET@Home Text Messages

REMINDER	Your first packet “Basics of Kidney Disease” is headed your way! Keep an eye out for the postcards for this packet as well.
MOTIVATION	You can slow down how fast your kidneys fail. Learn more about all of your options!
FACT	Did you know that the kidneys help keep bones healthy?
RECOMMENDATION	Chronic kidney disease (CKD) can present itself through a variety of symptoms. It’s important that you familiarize yourself with these symptoms so you can track changes and talk to your doctor.
QUIZ QUESTION	Can you slow down how fast your kidneys fail? Reply with YES or NO.
ANSWER FOR YES	That’s right! You can slow down how fast your kidneys fail.

If patients have questions for their kidney and transplant providers, they are directed to contact the *ET@Home* Help line, which can connect them with their KPSC providers for more information.

### Involving social support network

Our general recommendation through the program will be for patients to involve their family and friends in learning and decision-making about transplant. The brochures also recommend that the patient watch the video with other people in their lives who help them make important health decisions, particularly the stories of living donors, and complete the decision aid with them.

### Variables

Before the intervention, a baseline patient survey will measure the following independent variables: participants’ demographic characteristics, socioeconomic derailers to pursuit of transplant, medical mistrust, health-related quality of life (HRQOL), health literacy, and the amount of previous transplant education they have received. It will also assess the following dependent variables: level of current transplant knowledge, current ability to make an informed decision about transplant, self-efficacy to pursue DDKT and LDKT, and steps taken toward transplant. After the intervention, a post-survey will measure key study dependent variables to assess change, including the level of current transplant knowledge, current ability to make an informed decision about transplant, self-efficacy to pursue DDKT and LDKT, and steps taken toward transplant. For patients randomized to receive the *ET@Home* education, it will also assess their experiences with the educational materials through a program evaluation assessment.

#### Outcome measures and research methodology

In order to verify and demonstrate impact on improving knowledge of the process, risks, advantages, and disadvantages of deceased and living donation among dialysis patients, an RCT will be conducted. In this RCT, 1200 CKD patients in the KPSC system will be randomized to one of two education conditions (patient-guided *ET@Home* vs. standard KPSC transplant education, 600 patients per condition). Changes in key outcomes will be assessed comparing patient data collected at baseline and six months post-baseline. The primary outcome measures include patients’ knowledge of CKD and the risks and benefits of transplant and the capacity to make an informed decision about transplant. Additionally, key secondary measures will be examined, including self-efficacy to pursue DDKT and LDKT and steps taken towards transplant.

Comparisons in the two educational conditions’ effectiveness will also occur for patients earlier in the CKD continuum, patients who speak Spanish, and non-White patients.

#### Methodology

Patients will be remunerated $20 with a gift card after completing a baseline survey. Most surveys will be completed online by the study participants directly or over the phone with a trained interviewer. Any mailed surveys will be returned to KPSC and entered into the study’s assessment database. A double-data entry method will be used to ensure accurate data entry. Separate databases will be created for each patient’s registration/contact information and assessment data. Each patient will be given a unique, arbitrary study identification number that can be used to associate their contact and registration information and assessment data. Once the 1200 surveys have been collected and entered, this data will be analyzed to determine baseline differences in transplant knowledge, informed decision-making, self-efficacy, and steps toward transplant between patients varying in race/ethnicity, primary language, and CKD stage before patients are given an intervention.

### Evaluation and statistical analysis plan

#### Power analysis

Based on the following assumptions, we estimated the sample size for the RCT. Using data from previous RCTs [4, 35], a mean knowledge change score of 1.0 points is estimated necessary to be detected among patients receiving *ET@Home* and standard KPSC education. Patients’ baseline transplant knowledge scores will be correlated with those of other patients from the same clinics [intraclass correlation coefficient (ICC) of 0.31]. The variance in knowledge change has both between- and within-cluster components, yielding a pooled standard deviation estimate of 4.0. Participant retention will be 50% at the 6-month assessment. Outcomes will be assessed using two-tailed statistical tests. Type 1 error rate (alpha level) will be set at 0.05. This study design will ensure power of 0.90. Power calculations for this trial are based on all of these factors, which yields a standardized effect size, accounting for clustering of approximately 0.257.

Power analyses based on changes in transplant knowledge, our primary study outcome, are presented. The study design and analyses were treated as a test of the difference between the mean knowledge score change of CKD patients in the *ET@Home* and standard KPSC education (control) conditions six months post-baseline. It is estimated that with 600 completed surveys (50% attrition), there will be a 90% power to detect a 0.25 effect size between two equal-sized groups (control and treatment groups). By stratifying recruitment and randomization by race/ethnicity (Asian, Black, Hispanic, White), primary language spoken (English vs. Spanish), and stage of CKD, according to the table below, this sample size will also have 90% power to detect 1 point differences in change on the transplant knowledge scale between the *ET@Home* and standard KPSC education conditions for these critical subgroups using interaction terms (e.g., treatment by race interaction, language by race interaction). Patients enrolled and randomized are shown in Table 3.

**Table 3:** Patients enrolled and randomized

Race/Ethnicity/Language	Stage of CKD			
	3^a^	4	5/ESRD	Total
*English-speaking Asian*	67	67	66	200
*English-speaking Black*	67	66	67	200
*English-speaking White*	67	67	67	200
*English-speaking Hispanic*	67	67	66	200
*Spanish-speaking Hispanic*	133	133	134	400
Total	400	400	400	1200

^a^ *CKD* 3 patient recruitment prioritized based on likelihood to progress

### Measures

#### Demographic and clinical characteristics

Patient demographics, including patients’ gender, age, race/ethnicity, preferred language, and CKD stage will be obtained from the KPSC electronic medical record.

#### Kidney transplant Derailers index

Measures common transplant derailers such as level of education, health literacy, financial stability, neighborhood/environmental safety, access to transportation, and social support. Health literacy will be assessed with two items measuring subjective health literacy that were shown to detect health literacy as well as gold standard measures such as the S-TOHFLA and the REALM [36].

#### Global Health-related quality of life

Global HRQOL will be measured with the PROMIS 10-item global health scale [37], which produces summary physical and mental scores, each of which has 4 items supported by factor analyses and IRT analyses, with good reliabilities found: physical = 0.81, mental = 0.86.

#### Previous transplant education

The measure of previous transplant education captures the quantity and quality of transplant education, and it has been correlated with taking action toward pursuing transplant in previous studies [26].

#### Small steps

A measure of whether patients took any of 25 steps toward transplant (e.g., “Do you plan to talk through your treatment options with people you trust?” or “Do you plan to call the transplant center to begin evaluation?”). At each survey, patients will be asked whether they had “Already done,” “Plan to do,” or “Don’t plan to do” each step. These questions will be completed at baseline and six months.

#### Level of medical mistrust

Medical Mistrust will be measured with the Medical Mistrust Index (MMI) [38]. This scale was supported by factor analysis and exhibits moderately strong test-retest reliability, with correlation coefficients ranging from 0.3–0.7.

#### LDKT and DDKT self-efficacy

The factor structure for the LDKT scale was verified with exploratory and confirmatory factor analyses, demonstrating a good fit for a one-factor solution (loadings ranging between 0.61–0.91) [32]. The Cronbach’s alpha was 0.88, indicating a high level of internal reliability. The factor structure of the DDKT scale was also verified with exploratory and confirmatory factor analyses, demonstrating a good fit for a one-factor solution (loadings ranging between 0.50–0.73) [39]. The alpha was 0.85, indicating good internal consistency reliability. Self-efficacy will be measured at baseline and six months.

#### CKD, LDKT and DDKT knowledge

A knowledge scale was created using items our team has employed in several previous studies and legacy measures [6, 35, 40] and new questions in response to issues raised by patients and clinicians. A scale created from the extant items has been evidenced with factor analyses [41]. Knowledge will be measured at baseline and six months.

#### Informed decision-making

An informed decision-making measure was created from legacy measures and items generated from our formative work. Items for this scale were selected from legacy scales with good psychometric properties, like the Decisional Conflict Scale, whose total scale and subscales evidence high internal consistency, with Cronbach’s alphas ranging between 0.6–0.9. There was not a statistically significant difference between test-retest scores for this scale, and there was a high correlation (r = 0.80) between them [42]. Informed decision-making will be measured at baseline and six months.

#### Process evaluation

At the six-month follow-up assessment, all intervention participants will complete an evaluation of the helpfulness of and their satisfaction with the print and video materials, text messages, and the *ET@Home* program as a whole.

### Statistical analysis

The available demographic and clinical characteristics of patients who refuse to join the study will be compared to those who enroll to determine if the patient selection procedure has resulted in a biased sample. A similar analysis will be conducted to compare the patients who fail to complete the six-month assessment. Multiple imputation by chained equations (MICE) will be used to multiply impute missing data. Imputation models will include all analysis and stratification variables. The intent-to-treat (ITT) approach will be applied in that all study participants will be analyzed according to the group that they were randomized to, regardless of whether they actually received their assigned intervention or not. Responses to the six-month intervention evaluation will be summarized for the sample overall and by levels of the stratification factors.

It is hypothesized that patients earlier in the CKD continuum, patients who speak Spanish, and non-White patients will have less knowledge about the opportunities for and risks and benefits of living kidney donation and will be making less-informed LDKT decisions. Investigators will evaluate these hypotheses using data from the pre-survey (baseline) by fitting multilevel random effects models with a random intercept to account for clustering at the CKD clinic level to compare differences in knowledge, informed decision-making, and Self-Efficacy. First, unadjusted models will be examined with the race/ethnicity, primary language spoken, and stage of CKD as independent variables for each test (separate models for each). Then, each of these models will be adjusted for other factors hypothesized to impact each outcome, including level of socioeconomic vulnerability, the level of previous transplant education received, the level of medical mistrust, and the level of health literacy. The addition of these variables will help test and control for confounding.

Next, the effectiveness of *ET@Home* for CKD Stage 3–5 patients to increase LDKT knowledge and decision-making compared to KPSC education, by race/ethnicity and primary language spoken, will be evaluated. First, it will be determined if baseline differences in patient- and clinic-level variables are present using χ2 and Wilcoxon rank-sum tests, and subsequently adjustments for any out-of-balance characteristics in outcome analyses will be made. Differences in changes in transplant knowledge and Self-Efficacy using change scores, defined as the difference between the baseline and post-survey assessments, will be examined. Differences in these outcomes between the educational conditions will be tested using multilevel random effects models with normal outcome distributions, accounting for clustering with a random intercept for CKD clinic. Differences in informed decision-making will be examined using multilevel random effects logistic models. Finally, for the number of new steps taken, differences between the educational conditions in the count of new steps will be analyzed with a multilevel random effects Poisson model.

After the primary analyses are completed, multilevel random effects models will be constructed to examine the heterogeneity of treatment effect (HTE) using interaction terms for the *ET@Home* educational condition by the key patient subgroups by which randomization was stratified, including race, primary language spoken, and stage of CKD. For example, the *ET@Home* educational condition (*ET@Home* vs. SOC) x primary language (English vs. Spanish) interaction term will be tested, enabling us to determine of the *ET@Home* intervention was more or less effective for primarily Spanish-speaking patients than English-speaking patients.

## Discussion

Previous research has shown that improved transplant education, both inside and outside of transplant centers, can increase patients’ knowledge of DDKT and LDKT, pro-transplant attitudes, referral for transplant, and LKDT rates [20, 27–29]. However, this RCT will allow us the opportunity and ability to partner with KPSC, a large healthcare system with a diverse patient base, to compare *ET@Home* with their current educational standard-of-care. We also will be able to assess a significant amount of data from a fully integrated care management program, diverse membership, and ability to track a large patient population as they make treatment decisions along the most coordinated care system in the country.

The four-part modular *ET@Home* program, delivered to CKD 3–5 patients through video, print, and texting systems over six months, is one of the first examinations of supplementary education that does not involve healthcare providers. This intervention also applies health literacy and transplant education best practices recommended for programs outside of transplant centers to deliver education directly to patients [34, 43, 44].

In addition, this RCT will examine the effectiveness of this program on patients of various races, stages of CKD, and primary languages spoken. The findings of this trial will foster greater understanding of how *Explore Transplant@Home* specifically affects White, Black and Hispanic patients’ LDKT decision-making, knowledge, and behavior. Additionally, as it will serve patients whose primary language is English and Spanish, it will allow us to learn more about their interest in preemptive transplant, DDKT, and LDKT. We hope this program will reduce disparities in access to transplant for patients who speak Spanish and for Black, Hispanic, and Asian patients served by KPSC.

Previous studies have identified low SES as a barrier to transplant, with patients of low SES being less likely to be wait-listed [45, 46]. *Explore Transplant@Home* is unique in its ability to study the impact of SES on patients’ pursuit of LDKT. While previous studies have focused on SES factors that are difficult to modify [13, 47] or on the neighborhood or census block level [16, 47, 48], *ET@Home* will assess key, specific SES barriers and provide support services to address these barriers. This study will allow us to identify the most common SES-related influences challenging completion of transplant evaluation and getting an LDKT.

In conclusion, through this trial, investigators will gain an understanding of key knowledge gaps for patients along the CKD continuum and between patients who speak different languages. An RCT examining the effectiveness of both English- and Spanish-language *ET@Home* programs in increasing KPSC patients’ knowledge about the opportunities for, and risks and benefits of, LDKT and making informed transplant decisions will be completed. Investigators will have a clear understanding of the facilitators and barriers occurring within the KPSC system to the delivery of transplant and LDKT education for patients of varying racial and socioeconomic groups. If successful, the dissemination approach of *ET@Home* could easily and immediately be expanded to other CKD patients within KPSC and Kaiser Permanente Northern California. The lessons learned and approach to disseminating this low-cost *ET@Home* educational approach could be utilized within other healthcare and insurance companies, serving hundreds of thousands of diverse patients to assist in their informed decision-making for DDKT and LDKT before and after their kidneys fail. We hope this *ET@Home* program will help to reduce disparities in access to transplant and creates a model to be replicated for other healthcare systems.

## Abbreviations

AST: American Society of Transplantation
CKD: Chronic kidney disease
DDKT: Deceased donor kidney transplant
ESRD: End-stage renal disease
*ET*: *Explore Transplant*
*ET@Home*: *Explore Transplant@Home*
HRQOL: Health-related quality of life
HRSA: Health Resources and Services
ICC: Intraclass correlation coefficient
KPSC: Kaiser Permanente Southern California
LDKT: Living donor kidney transplant
MMI: Medical Mistrust Index
RCT: Randomized control trial
RRT: Renal replacement therapy
TTM: Transtheoretical Model of Behavioral Change
UNOS: United Network for Organ Sharing
